# Transcriptional Responses of Glutathione Transferase Genes in *Ruditapes philippinarum* Exposed to Microcystin-LR

**DOI:** 10.3390/ijms16048397

**Published:** 2015-04-15

**Authors:** Bruno Reis, Mariana Carneiro, João Machado, Joana Azevedo, Vitor Vasconcelos, José Carlos Martins

**Affiliations:** 1 CIIMAR/CIMAR—Interdisciplinary Centre of Marine and Environmental Research, University of Porto, Rua dos Bragas 289, 4050-123 Porto, Portugal; E-Mails: brunobuendia@gmail.com (B.R.); marianarcarneiro@gmail.com (M.C.); jprmachado@gmail.com (J.M.); joana_passo@hotmail.com (J.A.); vmvascon@fc.up.pt (V.V.); 2 Department of Biology, Faculty of Sciences, University of Porto, Rua do Campo Alegre, 4069-007 Porto, Portugal

**Keywords:** microcystins, glutathione transferases, real-time PCR, *R. philippinarum*, detoxification, biomarker

## Abstract

Glutathione Transferases (GSTs) are phase II detoxification enzymes known to be involved in the molecular response against microcystins (MCs) induced toxicity. However, the individual role of the several GST isoforms in the MC detoxification process is still unknown. In this study, the time-dependent changes on gene expression of several GST isoforms (pi, mu, sigma 1, sigma 2) in parallel with enzymatic activity of total GST were investigated in gills and hepatopancreas of the bivalve *Ruditapes philippinarum* exposed to pure MC-LR (10 and 100 µg/L). No significant changes in GST enzyme activities were found on both organs. In contrast, MC-LR affected the transcriptional activities of these detoxification enzymes both in gills and hepatopancreas. GST transcriptional changes in gills promoted by MC-LR were characterized by an early (12 h) induction of mu and sigma 1 transcripts. On the other hand, the GST transcriptional changes in hepatopancreas were characterized by a later induction (48 h) of mu transcript, but also by an early inhibition (6 h) of the four transcripts. The different transcription patterns obtained for the tested GST isoforms in this study highlight the potential divergent physiological roles played by these isoenzymes during the detoxification of MC-LR.

## 1. Introduction

The frequent eutrophication of water bodies creates the conditions for the development of cyanobacterial blooms which are characterized by excessive proliferation of cyanobacterial cells [1]. Cyanobacteria can produce potent and environmentally persistent toxins like microcystins (MCs) affecting not only freshwater and estuarine systems, but also marine habitats (land-sea flows). MCs are cyclic peptides consisting of seven amino acids including a unique β-amino acid side-group Adda, which is characteristic of cyanobacteria. More than 90 structural variants of MCs have been found, with variation occurring mainly at the two l-amino acids. MC-LR is a structural variant characterized by the presence of leucin (L) and arginin (R) as l-amino acids in positions 2 and 4 [2,3,4]. MC-LR main mechanism of toxicity in animals comprises the inhibition of several serine/threonine (Ser/Thr) protein phosphatases (PPs) [5,6] leading to increased phosphorilation of cellular proteins involved in signal transduction. Defective phosphorilation/dephosphorilation regulatory mechanisms leads to cytoskeleton disorganization and the disruption of cell integrity [4,7]. This effect has already been described in several organisms namely fish [8] and mammals [9]. MCs uptake is also known to increase oxidative stress in cells. The production of reactive oxygen species (ROS) [10] leads to an increase in lipid peroxidation [11,12], DNA damage [13] and alteration of the antioxidant defense system [11,14,15] with the depletion of intracellular glutathione (GSH). However, living organisms have developed different strategies to deal with the stressful conditions caused by MCs exposure. Detoxification enzymes such as glutathione transferases (GSTs) are in the first line of molecular defenses against environmental stressors. GSTs are a family of phase II dimeric enzymes [16] which catalyze the nucleophilic conjugation of GSH to various toxic exogenous and endogenous electrophiles to form more soluble metabolites. As a result, these substrates are rendered less toxic and capable of being eliminated through a GSH-conjugate recognizing transporter [17]. The existence of MC-LR-GSH conjugates formed through soluble GSTs activity was reported by Pflugmacher *et al.* [18] in several aquatic organisms including bivalves. Apart from direct detoxification of exogenous compounds, GSTs also have the ability to detoxify a number of harmful by-products formed due to stressful conditions. Normally, exposure to MC-LR leads to an increased production of ROS (superoxide anion O_2_^−^, hydrogen peroxide H_2_O_2_ and the hydroxyl radical OH) that can result in the formation of toxic carbonyl-, peroxide- and epoxide-containing metabolites within the cell. GSTs are able to detoxify these by-products of oxidative stress turning them harmless [16,17]. MCs have been found to accumulate in bivalve tissues at concentrations several times higher than concentrations found in water due to bivalve ability to filter large quantities of particles [19,20]. For this reason, suspension-feeding bivalves are considered efficient toxin vectors along food chains and can pose a risk to higher trophic levels through their consumption, possibly leading to livestock and human poisoning [7,21]. These organisms are also abundant in estuaries where much human contact with the aquatic environment occurs and are capable of withstanding baseline levels of pollution, showing relative insensitivity to toxicants compared to other aquatic organisms. These facts highlight the potential role of detoxification enzymes such as GSTs in bivalve resistance. Although many novel GST classes have been identified and classified from non-mammalian organisms, information on bivalve GSTs is still scarce. In this study, we aimed to investigate the *in vivo* toxic effects of dissolved MC-LR on GST gene expression in the estuarine clam *Ruditapes philippinarum*. To achieve such goal, we have investigated alterations at the transcriptional level of GST sigma 1, sigma 2, pi and mu gene expression in parallel with GST enzymatic activity in both hepatopancreas and gills in an exposure assay using purified MC-LR.

## 2. Results and Discussion

MCs have been shown to have a significant impact on aquatic organisms [22]. Among these, the bivalve mollusks, as sessile filter-feeding organisms, are especially susceptible to cyanotoxins intoxications. However, little is known about the biochemical mechanisms involved in the defense and toxicity to common toxins such as MCs in these mollusks. MCs as well as metals, bacterial endotoxins (LPS) or prooxidants like H_2_O_2_ have been found to induce oxidative stress in animal cells [23,24,25,26]. GSTs are a group of fundamental enzymes that protect organisms against the destructive effects of ROS and maintain cellular homeostasis by regulating the excess ROS levels [25]. In addition, GSTs can also detoxify MCs and other xenobiotics by directly conjugating the toxin with GSH. In this way, GST activity towards 1-chloro-2,4-dinitrobenzene (CDNB) has been widely investigated to evaluate GSTs induction upon organism exposure to toxic compounds. However, most of the experiments carried out so far, only measured total GST activity using CDNB as a substrate not accounting for substrate specificity from different GST isoforms. In previous studies with bivalves, the mRNA expression of specific GST genes have been investigated in organisms exposed to environmental pollutants, rendering information about a possible correlation between specific GST gene expression and the detoxification of particular contaminants. This information can be useful to disclosure which GST isoforms have significant roles in MC-LR detoxification. The current work evaluates the relative changes of gene expression of the different GSTs isoforms in mollusk bivalves exposed to MCs. The time-dependent transcriptional responses of *R. philippinarum* cytosolic GST isoforms and total activity were evaluated after exposure to 10 and 100 µg/L of pure MC-LR. These concentrations fall in the range of values found in natural waters, that can go from trace concentrations up to 1800 µg/L or higher, immediately after the collapse of a highly toxic bloom [27]. The clam *R. philippinarum* is an invasive species from the Indo-Pacific region introduced in European coastal waters for commercial farming in the 1970s [28]. It is an economically important species, widely distributed in middle to low intertidal zones in bays and estuaries and due to their habitat and filter-feeding mechanism, these organisms are recurrently exposed to toxic contaminants and natural toxins. In addition, *R. philippinarum* competes directly with the European native species for the same habitat and resources. After, this species introduction in Europe a massive decrease of the native clam (*Ruditapes decussatus*) has been registered in result of its higher growth rates and higher resistance to physical stress and pathogens [29,30]. Figueira and Freitas [28], reported that *R. philippinarum* collected from Ria de Aveiro estuary accumulated less 20% to 70% of contaminants than the native species. Furthermore, when the clams were subjected to depuration *R. philippinarum* reduced more the contaminant burden than *R. decussatus*. These facts highlight the potential role of detoxification enzymes such as GSTs in the adaptive and defensive responses of the clam *R. philippinarum*.

### 2.1. MC-LR Uptake by R. philippinarum

**Figure 1:**
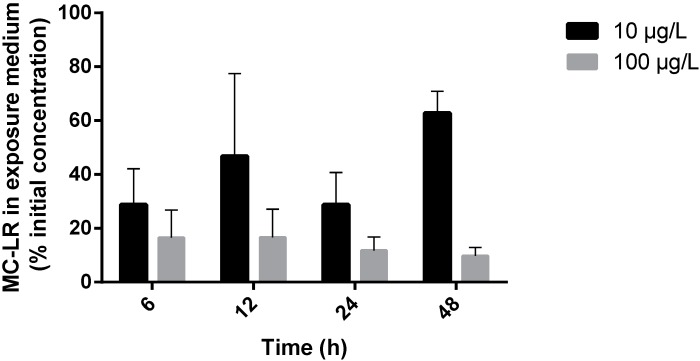
MC-LR in seawater (% of initial exposure concentration), taken from the flasks where the animals were exposed at the end of each experimental period. Black columns represent low-exposure group (10 µg/L MC-LR), light grey columns represent high-exposure group (100 µg/L MC-LR) (bars show standard deviation).

Marine bivalves are recognized for the rapid uptake and accumulation of cyanobacterial toxins [19,31]. Miller *et al.* [32] reported that *R. philippinarum* exposed to MC-LR showed high toxin accumulation in hepatopancreas, with tissue concentration approximately 100 times higher than exposure medium at 24 h. In this study, MC-LR concentration was determined in the exposure medium at each time of exposure (Figure 1).

According to previous studies, MCs are stable both in freshwater and seawater samples for several days [33,34]. In fact, Mazur and Plinski [34] assessed MC-LR stability in seawater samples taken from the ocean and found that at 24 h post-toxin addition the decomposition was close to zero. Our results point that for MC-LR exposed groups there was an uptake of the toxin, supported by the general decrease of MC-LR concentration in the exposure medium of both treatments (37% to 71% reduction in low-exposure; 83.4% to 90.2% reduction in high-exposure) at all exposure periods (Figure 1). Low-exposure groups (10 µg/L MC-LR) showed the lowest values of MC-LR in seawater at 6 and 24 h (29%) and the highest value was found at 48 h (63%). High-exposure groups (100 µg/L MC-LR) showed a less marked variation for the various experimental periods. From 6 to 48 h, MC-LR in seawater varied from 16.6% to 9.8% (Figure 1). However, differences between experimental times were not significant (*p* > 0.05) for each dose group.

### 2.2. Enzyme Activity Measurements

**Figure 2:**
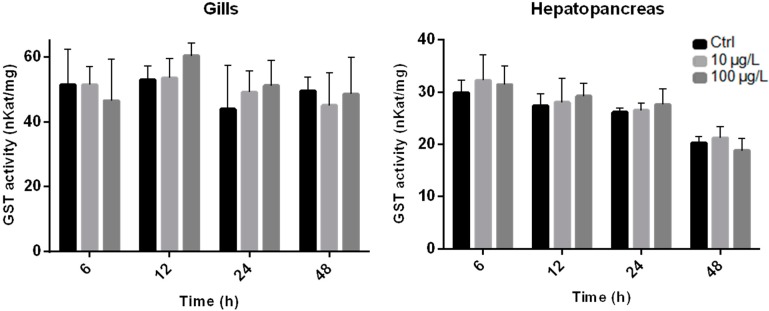
Total Glutathione Transferase (GST) activity (nKat/mg of protein) in *R. philippinarum* hepatopancreas and gills exposed to pure MC-LR over a period of 48 h. Black columns represent Control (Ctrl) group, light grey columns represents low-exposure group (10 µg/L MC-LR) and dark grey columns represent high-exposure group (100 µg/L MC-LR) (bars show standard deviation).

GSTs enzymes are expressed in a tissue specific manner and both gills and hepatopancreas were found to be the major organs of detoxification in bivalves [35,36,37] accounting for most of GST activity [38]. GST total activity was measured in both these organs of *R. philippinarum* exposed to 10 and 100 µg/L of purified MC-LR (Figure 2). Regarding tissue distribution, GST activity of the clams towards CDNB was found to be higher in gills (43.9 to 60.3 nKat/mg protein) than hepatopancreas (18.8 to 32.2 nKat/mg protein) for all exposure periods. None of the tested MC-LR concentrations induced any significant effects (*p* > 0.05) in GST total activity for both organs, although, a dose dependent increase is perceived for both at 12 and 24 h post-exposure (Figure 2). Recently, the zebramussel *D. polymorpha* exposed to a crude extract (10 and 50 µg/L) and pure MC-LR (10 and 50 µg/L) also did not show significant differences in GST activity in the hepatopancreas and gills after 24 h [39]. Still, when total GST activity in whole body tissue was measured, activity was significantly increased for both concentrations of the crude extract, whereas only the highest concentration of pure MC-LR caused an effect. The results of the present study together with those available in the literature demonstrate that MC-LR can exert different GST response, depending on both the animal tissues and species that are considered. In addition, differences between studies may be due to the complexity of the mixtures found on crude extracts as opposed to the single chemical exposure when using pure MC-LR. Vasconcelos *et al.* [37] found that a cyanobacterial crude extract elicited higher GST activity in several organs of *M. galloprovincialis* compared to pure MC-LR.

Different response patterns, like those induced by MCs in GST total activity, are also seen when organisms are exposed to other xenobiotics. For example, in a laboratory experiment mussels (*M. galloprovincialis*) exposed to 100 µg/L of benzo[α]pyrene (B[α]P), showed no significant changes on GST activity in hepatopancreas [40]. However, Gowland, *et al.* [41] found that GST activity was increased in the same organ of *Mytilus edulis* exposed to heavy loads of polycyclic aromatic hydrocarbons (PAHs). In addition, in *R. decussatus* no significant changes were reported on GST activity in the hepatopancreas when these animals were exposed to organochlorine compounds. In contrast, the same authors found that GST activity increased in gills [36].

### 2.3. Gene Expression

**Figure 3:**
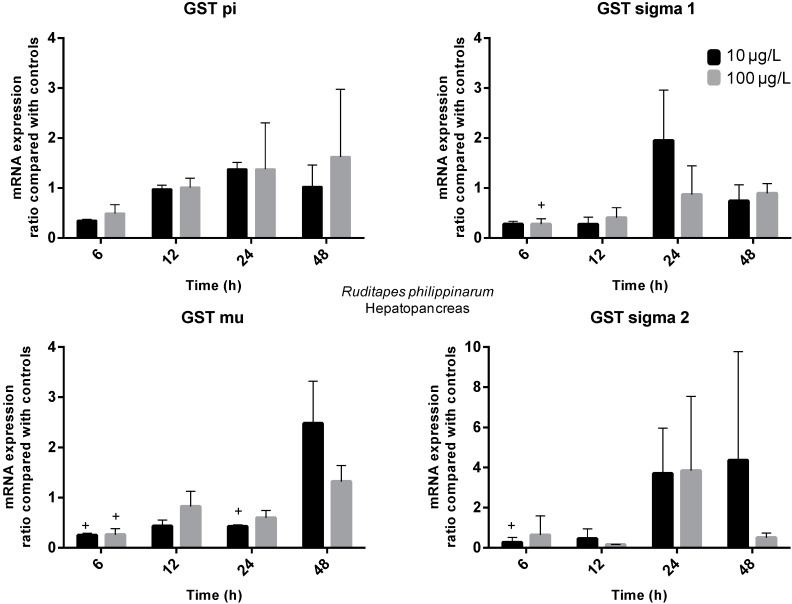
Temporal changes of *R. philippinarum* hepatopancreas GSTs transcripts after pure MC-LR exposure compared with controls (Treatment group ratio/Ctrl ratio). Black columns represent low-exposure group (10 µg/L MC-LR) and grey columns represent high-exposure group (100 µg/L MC-LR) (statistically significant differences were accepted at *p* ≤ 0.05; + indicates differences to Ctrl).

**Figure 4:**
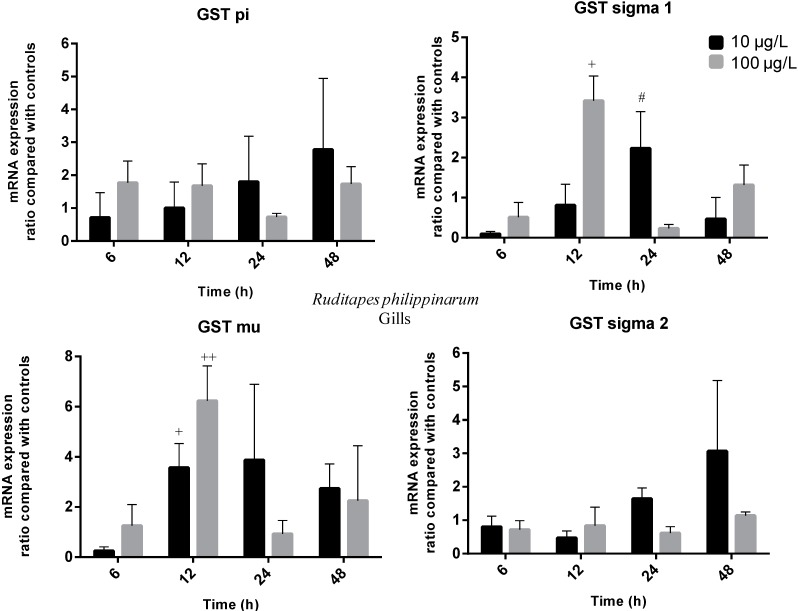
Temporal changes of *R. philippinarum* gills GSTs transcripts after pure MC-LR exposure compared with controls (Treatment group ratio/Ctrl ratio). Black columns represent low-exposure group (10 µg/L MC-LR) and grey columns represent high-exposure group (100 µg/L MC-LR) (statistically significant differences were accepted at *p* ≤ 0.05; + indicates differences to Ctrl; ++ indicates differences both to Ctrl and low-exposure group; # indicates differences between exposed groups).

Transcriptional activity allows the assessment of individual GST genes encoding different GST isoforms. Previous studies with aquatic organisms showed that MCs induced variations in the transcription of many GST isoforms [42,43,44]. In the present work, a diverse variation in gene transcription of several GST isoforms (pi, mu, sigma 1 and sigma 2) was reported on the gills and hepatopancreas of *R. philippinarum* exposed to 10 and 100 µg/L of MC-LR. The time-dependent transcriptional changes of GST mRNAs in both organs of the clams are shown in Figure 3 and Figure 4.

In hepatopancreas, the transcription of all GST isoforms decreased at 6 h post-exposure (between 3.8- and 5.7-fold). This decrease was significant (*p* ≤ 0.05) in both exposure groups for GST mu gene and in high and low-exposure groups for sigma 1 and 2 genes, respectively. GST pi, sigma 1 and sigma 2 transcription recovered to control levels after 12 h in both exposure groups. In fact, the transcription of GST sigma 1 increased (two-fold) for low-exposure group at 24 h post-exposure, although not significantly (*p* > 0.05). The same increase trend was detected in GST sigma 2 transcription for both exposure groups (between 3.7- and 3.9-fold) at 24 h and for the low-exposure group (4.4-fold) at 48 h (*p* > 0.05). Interestingly, the GST mu transcription evidenced a 2.4-fold decrease (*p* ≤ 0.05) at 24 h post-exposure for the low-exposure group. In contrast, transcription of the same isoform was 2.5-fold higher (*p* ≤ 0.05) after 48 h exposure to MC-LR for the same dose group. In gills, transcription of GST pi was relatively stable throughout all experimental time for the high-exposure group. In contrast, an apparent increase trend is perceived at 24 (1.8-fold) and 48 h (2.8-fold) post-exposure for the low-exposure group (*p* > 0.05). The transcription of GST sigma 2 increased (3.1-fold) at 48 h post-exposure for the low-exposure group (*p* > 0.05), similarly to hepatopancreas. A 3.4-fold increase (*p* ≤ 0.05) of GST sigma 1 transcription level was detected at 12 h post-exposure for the high-exposure group. For the same isoform, major changes on GST gene transcription (2.2-fold increase) after 24 h are attributable to low-exposure group, in the same way as in hepatopancreas. The transcription of GST mu isoform increased 3.6- and 6.2-fold for low and high-exposure groups, respectively, at 12 h post-exposure (*p* ≤ 0.05). This gene transcription levels remained up-regulated (24 h: 3.9-fold, 48 h: 2.7-fold) for low-exposure group throughout the remaining experimental time points (*p* > 0.05).

Similar results have been found for these four GST isoforms in previous studies with MCs or other xenobiotics. For example, Li *et al.* [44] assessed GST pi expression in a freshwater fish species (*Carassius auratus* L.), and found that expression was decreased in liver after exposure to 50 µg MC-LReq/kg BW. In our experiment, the transcription of GST pi isoform decreased in hepatopancreas at 6 h post-exposure, although not significantly (*p* > 0.05). Zhang *et al.* [45] evaluated GST sigma isoforms 1 and 2 mRNA expression in *R. philippinarum* hepatopancreas exposed to PAHs and metals. Exposure to B[α]P (5 and 50 μg/L) promoted a decrease of GST sigma 1 expression at 24 and 48 h post-exposure, while GST sigma 2 displayed increased expression levels from 24 to 96 h post-exposure. Regarding metal contamination, GST sigma 1 remained close to control levels while GST sigma 2 was significantly down-regulated at 24 and 48 h upon Cd exposure (10 and 40 μg/L). However, both genes were induced by copper (Cu) exposure (10 and 40 μg/L) from 24 h till the end of the experiment. Our data shows that in hepatopancreas both GST sigma isoforms 1 and 2 mRNA transcripts are down-regulated at 6 h post-exposure and even though differences are not significant, transcription tend to increase at later stages of exposure (24 and 48 h post-exposure). In gills, GST sigma 1 was found to be strongly up-regulated at 12 h (3.4-fold). Umasuthan *et al.* [26] injected *R. philippinarum* with bacterial lipopolysaccharide (LPS) and evaluated GST sigma gene expression in gills. Results show a significant up-regulation (3.7-fold) at 3 h post-injection and a similar induction pattern was also detected at 12 and 48 h post-exposure. Our results showed that GST mu transcription in the hepatopancreas was decreased at 6 h post-exposure but at 48 h the exposure to 10 µg/L MC-LR caused a 2.5-fold increase, significantly inducing gene transcription. In goldfish (*C. auratus*) exposed to a cyanobacterial crude extract (50 and 200 MC-LReq µg/Kg BW) GST mu gene expression was induced in the liver. Temporal changes were found to be dependent on MC-LR concentration as gene expression increased at early stages in the low dose group and from 12 to 24 h in the high dose group [43]. Furthermore, in *R. philippinarum* hepatopancreas, GST mu mRNA transcription levels showed significant increases (2–4.8-fold) after exposure to Cu at 24 and 48 h post-exposure [45]. In gills we found that GST mu transcription was highly up-regulated at 12 h post-exposure with the high exposure treatment causing a 6.2-fold increase. GST mu mRNA transcription induction in the gills was also reported by Bathige, *et al.* [46] when *R. philippinarum* was challenged with LPS. These authors reported that the expression profile of GST mu revealed elevated levels in a time dependent manner. The highest fold change was found at 3 h post-challenge but levels remained elevated till 12 h post-exposure and again at 48 h.

Overall, our results showed that MC-LR exposure caused differential expression of the four GST isoforms in hepatopancreas and gills of *R. philippinarum*. GST transcriptional changes in gills promoted by MC-LR are characterized by an early (12 h) induction of mu and sigma 1 transcripts. On the other hand, the GST transcriptional changes in hepatopancreas are characterized by a later induction (48 h) of mu transcript, but also by an early inhibition (6 h) of the four transcripts. Individually, transcriptional patterns promoted by MC-LR exposure for the several GST isoforms varied not only within each organ but also between organs. The gills, as an external organ are in direct contact with the environment and possibly reflect short term exposure, whereas the hepatopancreas, being an internal organ where xenobiotics can accumulate, reflects long term exposure [40,46]. The different transcription patterns obtained for the tested GST isoforms in this study highlight the potential divergent physiological roles played by these isoenzymes during the detoxification of MC-LR. GST mu and sigma 1 showed similar gene expression profiles in gills and accounted for major changes characterized by up-regulation at 12 h. In addition, in hepatopancreas both genes show gene expression inhibition at 6 h post-exposure. These genes seemed to be more sensitive to MC-LR exposure in both organs. It has been suggested that mu and sigma class GSTs participate in cellular xenobiotic defenses and in the metabolism of products of oxidative stress [25,45,46] in different organisms.

In this study, we reported transcriptional changes at different time points both in hepatopancreas and gills, but we did not found differences on enzyme activity for both organs. Regarding what happens *in vivo*, according to Amado and Monserrat [23], inhibition of protein phosphatases leads to a cellular hyperphosphorylated state that promotes the phosphorylation of transcription factors (Nrf2) involved in the expression of phase II detoxifying enzymes [47]. Phosphorylation increases Nrf2 mean life favoring the transcription of genes involved in antioxidant response, such as GST and glutamate cysteine ligase (GCL) the rate limiting enzyme in glutathione biosynthesis [48]. At the same time, phosphorylation inhibits GCL activity [49], promoting the continuous reduction of GSH, while activating GSTs [50,51]. However, low GSH concentration impairs GST capability to conjugate electrophilic compounds to GSH. Given this, when animals are exposed to MCs it is possible that even though transcription is up-regulated, GST activity might not increase. However, when measuring GST activity, GSH is not limiting, but rather supplied to the reaction. A possible explanation for the lack of enzyme activity changes in this study could be related to the usage of the universal substrate CDNB. As a result of that, some GST isoforms can be underestimated in the representation of total GST activity within an organism. GSTs show high catalytic promiscuity turning it difficult to define a distinction between different classes through specific substrate because of broad and overlapping substrates.

In addition, a dose dependent effect was not clear between low and high exposure groups. In the same way Li *et al*. [44] exposed *Carassius auratus* to a cyanobacterial crude extract (50 and 200 MC-LReq µg/kg BW) and did not establish such an effect. On the other hand, Xu *et al.* [52] reported a cumulative effect on *Ruditapes philippinarum* between GST pi expression and increasing concentrations of B[α]P. MCs toxicity depends on the balance and dynamics between accumulation and metabolism [53].

## 3. Experimental Section

### 3.1. Test Species and Cultures

For laboratory studies *R. philippinarum* were purchased from a local producer (Conchamar, Foz do Arelho, Portugal). Animals were acclimated to laboratory conditions in a 200 L storage tank with aerated seawater at 16 ± 1 °C and 33‰ salinity for 3 days, prior to exposure experiments. The water was changed every day and the animals were fed with an algal suspension (*Chlorella vulgaris*, 1 × 10^5^ cells·mL^−1^) every other day. *Microcystis aeruginosa* (strain 91094 from LEGE library) was cultured in 10 L flasks containing 8 L of Z8 medium, using cool white fluorescent light (10 μmol·m^−2^·s^−1^) with a light-dark period of 14–10 h, and a temperature of 25 ± 1 °C.

### 3.2. MC-LR HPLC Analysis

#### 3.2.1. Microcystin-LR Extraction for Analytical and Semi-Preparative HPLC

After achieving the desired cell density, *M. aeruginosa* biomass was collected through centrifugation at 4600× *g* for 10 min at 4 °C. The supernatant was discarded and the pellet was ressuspended in methanol (MeOH, HPLC grade) (50%), which was used as the extraction solvent. The sample was then sonicated, in an ice bath, at 60 Hz for 5 × 1 min. (VibraCell 50-sonics & Material Inc., Danbury, CT, USA). The homogenate was centrifuged at 4600× *g* for 15 min to remove cell debris at 4 °C. The resulting supernatant was then collected and the pellet was re-extracted following the same methodology. The supernatants resulting from both extraction steps were combined and stored at 4 °C. The concentrated MC-LR extract was thereafter purified and quantified by High Performance Liquid Chromatography with Photo-Diode Array (HPLC-PDA).

#### 3.2.2. MC-LR Purification

The MC-LR semi-preparative assay was performed using a reversed phase column (Phenomenex Luna RP-18 (25 cm × 10 mm, 10 μm)) kept at 45 °C. The isocratic elution was done with methanol 60% acidified with 0.1% trifluoroacetic acid (TFA) with a flow rate of 1.5 mL/min. The injected volume was 500–1000 µL. Peak purity and percentage of purified MC-LR was calculated at 214 and 238 nm.

#### 3.2.3. MC-LR Quantification

The MC-LR purified fractions were then quantified in the same HPLC system on a Merck Lichrospher RP-18 endcapped column (250 mm × 4.6 mm i.d., 5 µm) equipped with a guard column (4 mm × 4 mm, 5 µm) both kept at 45 °C. The PDA range was 210–400 nm with a fixed wavelength of 238 nm. The linear gradient elution consisted of (A) MeOH + 0.1% TFA and (B) H_2_O + 0.1% TFA (55% A and 45% at 0 min, 65% A and 35% B at 5 min, 80% A and 20% B at 10 min, 100% A at 15 min, 55% A and 45% B at 15.1 and 20 min) with a flow rate of 0.9 mL/min. The injected volume was 20 µL. The PDA range was 210–400 nm, with a fixed wavelength at 238 nm. The MC-LR purified sample was identified by comparison of spectra and retention time with a reference material of MC-LR (batch n° 018K1209, 10.025 µg/mL in MeOH, 98% purity, Cyano Biotech GmbH, Berlin, Germany). Sample purity was of 93%. The system was calibrated by using a set of 7 dilutions of MC-LR reference material (0.5 to 20 μg·mL^−1^) in methanol 50%. Each vial was injected in duplicate and every HPLC run series of ten samples was constituted with a blank and two different reference material concentrations in 50% methanol. Empower 2 Chromatography Data Software was used for calculation and reporting peak information. The minimum amounts (limit of detection) of MC-LR that can be detected in water its 0.2 μg·mL^−1^, based on a signal-to-noise ratio of 3. Limit of quantification of MC-LR in water is 0.5 μg·mL^−1^, based on a signal-to-noise ratio of 10. The retention time of the MC-LR peak was 10.44 min. All HPLC solvents were filtered (Pall GH Polypro 47 mm, 0.2 μm) and degassed by ultrasound bath.

### 3.3. Exposure Experiments

Each replicate consisted of three animals exposed in 700 mL of aerated seawater at 15 ± 1 °C and 34‰ salinity within 1 L glass flasks. Both controls and treatments were carried out in triplicate. The treatment groups were exposed to 10 and 100 μg/L of purified MC-LR mixed with *C. vulgaris* (1 × 10^5^ cells·mL^−1^) to prevent the inhibitory effect of MC alone. Tissues and medium sampling were done after 6, 12, 24 and 48 h of exposure to the toxin. The medium was daily renewed. Gills and hepatopancreas of the collected animals were manually dissected at 4 °C, weighed, pooled (3 individuals), frozen in liquid nitrogen and ground to a fine powder. These finely ground tissues were used for RNA (30 mg of tissue) and enzyme extraction (remaining tissue).

### 3.4. Toxin Analysis in Exposure Medium

Medium samples were filtered with a 0.45 µM filter and diluted with ultra pure H_2_O. Toxin quantification in exposure medium was made through an enzyme-linked immunosorbent assay (ELISA) (Microcystins-ADDA ELISA; Microtiter Plate; Abraxis, Philadelphia, PA, USA) with a detection limit of 0.1 µg/L. MC contents in medium are expressed in µg/L. 

### 3.5. Enzyme Activity Measurements

Each pool of ground tissue was homogenized on ice in sodium phosphate buffer (0.1 M, pH 6.5) containing 20% glycerol (*v*/*v*), 1.4 mM dithiothreitol (DTT), 1 mM Ethylenediaminetetraacetic acid (EDTA) and Halt protease inhibitor cocktail (Thermo Scientific, Hudson, NH, USA). Homogenization was made maintaining a constant ratio of 5 mL buffer per gram of tissue. Cell debris was removed by centrifugation at 9000× *g* for 30 min (4 °C) and the supernatant was stored at −20 °C until posterior use. GST activity was performed in the hepatopancreas and gills of bivalves exposed to MC-LR. GST activity with CDNB was measured as described by Habig, *et al.* [54] adapted to microplate, following the procedure described in Frasco and Guilhermino [55]. All assay incubations were conducted at 25 °C. CDNB was dissolved in ethanol, with the final reaction concentration less than 0.01%, and GSH was dissolved in buffer. For hepatopancreas and gill assays, the reaction mixture (300 µL final volume) contained 0.3 and 0.2 mg·prot/mL, respectively, along with substrate, GSH, and sodium phosphate buffer (0.1 M) with pH 6.5. Protein quantification was conducted using a microplate adapted protocol of the Bradford method [56] using bovine serum albumin as standard. Briefly, 5 µL of the diluted samples were mixed with 250 µL of Bradford reagent (B6916; SIGMA, St. Louis, MO, USA) and after 15 min incubation the absorvance was read at 595 nm. All determinations were conducted in triplicate on a temperature-controlled BioTek microplate reader (Synergy HT, 2009; BioTek Instruments, Winooski, VT, USA) in 96-well microplates.

### 3.6. Gene Expression

#### 3.6.1. RNA Extraction

Total RNA was extracted from exposed and control animals according to Qiagen’s RNeasy Mini Kit protocol for purification of total RNA from animal tissues. First, the pooled and grounded organs were transferred to a suitable vessel and 600 µL of homogenization buffer (RLT buffer) was added. Then, disruption and homogenization of the tissues were carried out using Precellys^®^ 24 tissue homogenizer (Bertin Technologies, Montigny le Bretonneux, France). The lysate was then centrifuged at full speed for 3 min and the supernatant was removed and transferred to a new vessel where it was mixed 1:1 with 70% ethanol. After this step, 700 µL of the sample was transferred to an RNeasy spin column, which was centrifuged for 15 s. at 8000× *g* and the flow-through was discarded. Then, 700 µL of RW1 buffer was added to the spin column, which was centrifuged as referred above. Afterwards, washing steps continued by addition of 500 µL of RPE buffer with the centrifugation conditions maintained as referred above. Before elution a last washing step was performed with 500 µL of RPE buffer and the column was then centrifuged for 2 min at 8000× *g* and the flow-through discarded. Finally, a total volume of 30 µL of sample was eluted using RNase-free water. A master solution containing Quant-IT reagent (1 µL × n samples) and Quant-IT working solution (199 µL × n samples) was prepared for quantification of RNA content of the samples. Afterwards, 190 µL of master solution plus 10 µL of Quant-IT broad range RNA were mixed to prepare the standards used to quantify sample RNA: Tubes containing 1 µL of sample RNA and 199 µL of master solution were prepared, vortexed and incubated for 2 min at room temperature. Finally, RNA concentration was measured photometrically with Qubit Fluorometer (Invitrogen, Carlsbad, CA, USA).

#### 3.6.2. cDNA Synthesis

Total cDNA for the real-time polymerase chain reaction (PCR) were generated from 1000 ng of total RNA from all samples according to nzytech’s first-strand cDNA synthesis Kit protocol. For each reaction it was Used 10 µL of NZYRT 2× Master mix (a mixture of reaction buffer, poly dT and random primers), 2 µL of reverse transcriptase, RNA template and Nuclease-free water until a total volume of 20 µL. The tubes were transferred to a PCR cycler (Biometra^®^ TGRADIENT, Göttingen, Germany). The reaction conditions were as follows: 10 min at 25 °C, 30 min at 50 °C and 5 min at 85 °C. Additionally, 1 µL of RNase H (*E. coli*) was added to the mixture and the reaction vessel was incubated for 20 min at 37 °C. The enzyme was inactivated by heating at 85 °C for 5 min.

#### 3.6.3. Primers Design

All primers were obtained from Invitrogen. Specific primers (Table 1) used for this study were taken from the following bibliography: Zhang *et al.* [45] (GST sigma 1, 2 and mu); Xu *et al.* [52] (GST pi). Specific primers were also designed for elongation factor 1-α (EF1-α) after obtaining *R. philippinarum* EF1-α sequence using specific primers designed for the flat oyster, *Ostrea edulis* [57]. The PCR products using the specific primers were sent for sequencing to confirm the specificity of the amplified products.

**Table 1:** Primer pair sequences and product length. Genes quantified through Real-Time PCR.

GST Gene	Primer Sequence (5'–3' Order)		Product Length (bp)
	Forward	Reverse	
sigma1	CAGAAGAATTTGGCAGAAGTAG	AAGACAGCAAGATCAGCGAG	121
sigma 2	AAGGCTAAACTTACAGAGGAG	GTGTTTCTTGAGTTCAGGGT	209
mu	GACTTCCCAATGTACGAGCTT	ACACTTTCCTGAGCGAGATAC	139
pi	GCATTACCGACCCTCAAAGC	CCATTGACGGGCATTTTCTT	101
EF1-α	GCTCACAGAAGCTGTACCAGG	CTGGGCATAGAAGCTTGCAG	136

#### 3.6.4. Quantitative RT-PCR

Quantitative RT-PCR was performed using a iCycler iQ™ Real-Time PCR Detection System (Bio-Rad, Hercules, CA, USA). The following genes were examined in the qPCR experiments: GST pi, mu, sigma 1, sigma 2 coding for GST pi, mu and sigma enzymes. EF-1 α was used as a control gene for DNA level normalization. The EF-1 α was previously used as reference gene in other studies [31,58]. Sample cDNA was 10-fold diluted with ddH_2_O. Each reaction mixture consisted of 2 and 4 μL of cDNA template for hepatopancreas and gills respectively, 0.25 μM of each primer; 1× IQ SYBR Green Supermix (Bio-Rad) and water to adjust to 20 μL final reaction volume. The 96 well plate was then transferred to a qPCR cycler (Biorad^®^ IQ™). The qPCR conditions for *R. philippinarum* genes and reference gene, were as follows: 95 °C of initial denaturation for 30 s; 40 cycles at 95 °C for 10 s, 60 °C (GST Sigma 1, 2, Mu and Pi) for 20 s and 72 °C for 20 s. A melting curve was generated for every run to confirm specificity of the assays. A cDNA pool of hepatopancreas and gills from Ctrl animals was used for normalization between qPCR runs. Efficiency tests were performed to examine the quality of PCR reaction and all assays showed efficiencies between 98.9% and 110%. Efficiency is an important parameter for the calculation of gene expression, it reports fold increase between cycles. Cycle Threshold data analyses were carried out according to Pfaffl method and following the next equation [59]:

(1) $$\text{R}=\frac{{\left({\text{E}}_{\text{target}}\right)}^{\Delta C\text{t target}(\text{control}-\text{treated})}}{{\left({\text{E}}_{\text{reference}}\right)}^{\Delta C\text{t reference}(\text{control}-\text{treated})}}$$

in which R = ratio, E = efficiency, ref = reference gene, and target = target genes. Cycle Threshold (*C*_t_) describes the cycle number, at which the fluorescence signal gains in strength exponentially. This signal relates to PCR products amplification, in samples with increasing cDNA template, *C*_t_ will decrease.

### 3.7. Statistical Analysis

All data was tested for normality and homogeneity of variances (data that showed non-normal distribution or had unequal variance was transformed to the natural logarithm) and then submitted to a one-way ANOVA. When these tests showed significance, individual means were compared using Tukey’s test. If data were either non-normally distributed or had unequal variances, Median test for K-samples was applied, when these tests showed significance Mann Whitney U-test was performed. For all statistical analyses, differences were regarded to be significant when *p* ≤ 0.05. All tests were performed with the statistical programme IBM SPSS STATISTICS, 20.0 package (IBM Corporation, New York, NY, USA).

## 4. Conclusions

A better understanding of GST gene expression on different organs is fundamental to improve the knowledge of which GST class is directly involved in the molecular response to MC exposure. GST transcriptional changes reflected more accurately the influence of MC-LR exposure on the GST detoxification system of *R. philippinarum* than total GST activity. Additionally, GST sigma 1 and mu showed the most significant changes in both organs and seemed to be more sensitive to MC-LR exposure. In this way, the transcription of GST sigma 1 and mu genes can potentially be used as a tool to assess MCs induced chemical stress in biomonitoring studies in *R. philippinarum*. In the future, further experiments are needed to better understand the regulatory mechanisms of these genes.
